# Middle Cerebral Artery Aneurysm and Distal Anterior Cerebral Artery (DACA) Aneurysms Related to Azygos and an Unusual Single Pericallosal Artery Variant: A Case Report

**DOI:** 10.7759/cureus.80219

**Published:** 2025-03-07

**Authors:** Victor R Chavez-Herrera, Pedro González Zavala, Eduardo Ichikawa-Escamilla, Jesus E Falcon-Molina, Joel A Velázquez Castillo, Blas E Lopez

**Affiliations:** 1 Neurosurgery, Centro Medico Nacional Siglo XXI, Mexico City, MEX; 2 Neurosurgery, Universidad Nacional Autonoma de Mexico, Mexico City, MEX

**Keywords:** aneurysm, anterior cerebral artery variant, daca, mirror aneurysm, pericallosal artery

## Abstract

The authors describe the case of multiple rare distal anterior cerebral artery (DACA) aneurysms related to a middle cerebral artery (MCA) aneurysm, an azygos, and an undescribed anterior cerebral artery (ACA) anatomical variation. A 61-year-old woman in a severe clinical state was diagnosed with subarachnoid hemorrhage (SAH) secondary to a ruptured anterior A3 DACA aneurysm. The patient also had unruptured kissing superior A3 DACA aneurysms, an atherosclerotic DACA aneurysm between the anterior and superior DACA aneurysms, and an unruptured MCA left aneurysm. Two pericallosal anatomical variations were seen: an A2 azygos and a rare single post-bifurcation (callosomarginal artery) pericallosal artery. All aneurysms were clipped using a single-staged left craniotomy with interhemispheric, subfrontal, and transsylvian access. This study demonstrates a patient with rare anatomical ACA variants and multiple complex aneurysms treated in a single-stage craniotomy.

## Introduction

Distal anterior cerebral artery (DACA) aneurysms are a rare site for aneurysm formation; it accounts for only 2-9% of all intracranial aneurysms (IA) [1]. DACA aneurysms (DACAa) require significant skill to treat due to their broad base, challenging access, and numerous anatomical variations [1]. It is well known that DACA aneurysms relate to multiple anterior cerebral artery (ACA) anatomical variations, mainly in the A2 segments of the ACA, such as azygos, trifurcation, and bihemispheric. It can also relate to multiple aneurysms (25-55%) and arteriovenous malformations [1-4]. Fisher’s segmentation of the five portions of the ACA usually specifies where the DACA aneurysms originate [5]. The publication by Lehecka et al. more precisely identifies the specific location [2]. DACA aneurysms typically arise in the A3 segment of the ACA, primarily at the junction of the callosomarginal and pericallosal arteries in the anterior A3 segment, representing 69-82% of the cases. Approximately 1% are situated in the superior A3 segment [1]. The azygos ACA is characterized by the absence of an anterior communicating artery (ACoA) and the presence of only one A2 DACA. This variant supplies irrigation to both frontal and parietal hemispheres medially and is present in only 0.05% to 1.8% of the population [4,6-9]. There are different terms and definitions for the azygos variant, and almost always, the evidence explicitly identifies it as only the A2 segment. Different publications have used the terms azygos pericallosal artery, unpaired pericallosal artery, unpaired cerebral artery, and common arterial cerebral trunk. True post-bifurcation (callosomarginal) single pericallosal trunk is extremely unusual [10,11]. The azygos variant has a strong relationship with aneurysm formation, which is seen in 9-71% of the patients [12]. Furthermore, bilateral, also known as mirror or “Kissing” type DACA aneurysms, are rare and have been scarcely published, with a prevalence ranging from 0.2% to 0.9% [1,13-15].

We present a 61-year-old female patient with subarachnoid hemorrhage (SAH) secondary to four DACA aneurysms (giant callosomarginal bifurcation anterior A3, frontal medial branch, and “kissing” superior A3) and a left middle cerebral artery (MCA) aneurysm with an azygos A2 variant and a single A3 post-bifurcation (callosomarginal) trunk that required clipping. To our knowledge, no publications have described a single A3 DACA post-bifurcation trunk leading to superior A3 DACA “kissing” aneurysms [2,4,6-8,16,17]. The presence of the post-bifurcation A3 single trunk pericallosal artery, combined with the occurrence of mirror aneurysms at the superior A3 DACA segment, as well as an anterior A3 DACA aneurysm and an additional left MCA aneurysm, all treated within a single surgical event, renders this an extremely rare case.

## Case presentation

**Figure 1:**
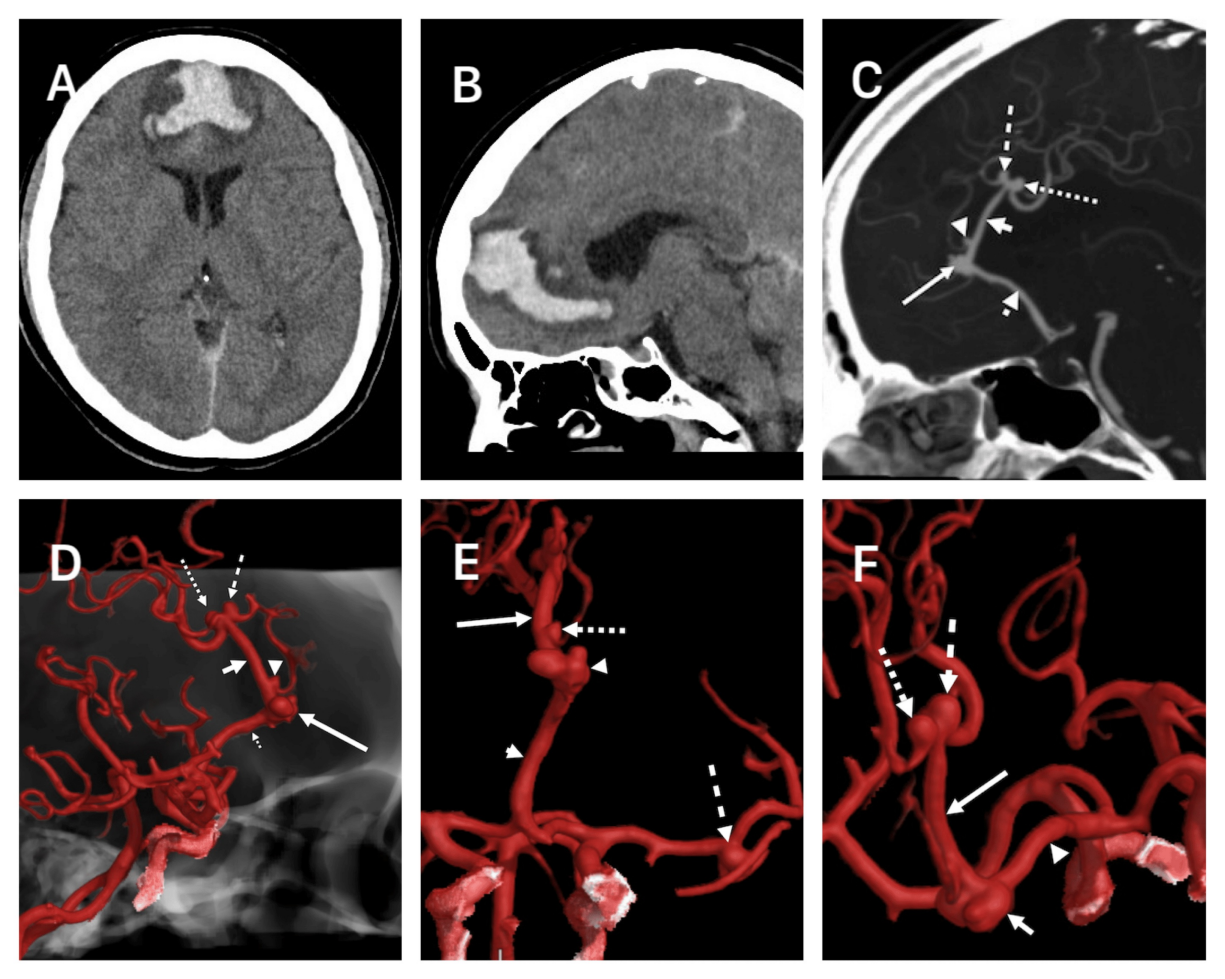
CT images and 3D reconstructed angioCT. (A) Axial CT showing an interhemispheric-frontal hematoma. (B) Sagittal CT showing the interhemispheric hematoma extending subcallosally. (C) Sagittal AngioCT evidencing four DACAa, an anterior A3 (large arrow), two “kissing” superior A3 (dashed and dotted arrows), and a small A3 between the anterior and superior A3 (arrowhead); azygos A2 ACA variation (dotted arrowhead) and the single A3 pericallosal artery ACA variation (small arrow). (D) Lateral angioCT reconstruction showing multiple DACAa, the large anterior A3 (long arrow), “kissing” superior A3 (long dashed and dotted arrows), small A3 (arrowhead), azygos (short dotted arrow), and the single pericallosal A3 artery (short arrow). (E) Anteroposterior angioCT showing the azygos (short arrow), single pericallosal artery A3 trunk (long arrow), and three aneurysms, the large anterior A3 DACA (arrowhead), the small A3 DACA (dotted arrow), and the MCA (dashed arrow). (F) Superior view angioCT of the “kissing” superior A3 DACAa (dashed and dotted arrows), single A3 pericallosal artery (long arrow), large lobulated anterior A3 DACAa (short arrow), and the azygos variant. CT: computed tomography, DACAa: distal anterior cerebral artery aneurysm, ACA: anterior cerebral artery, MCA: middle cerebral artery.

**Figure 2:**
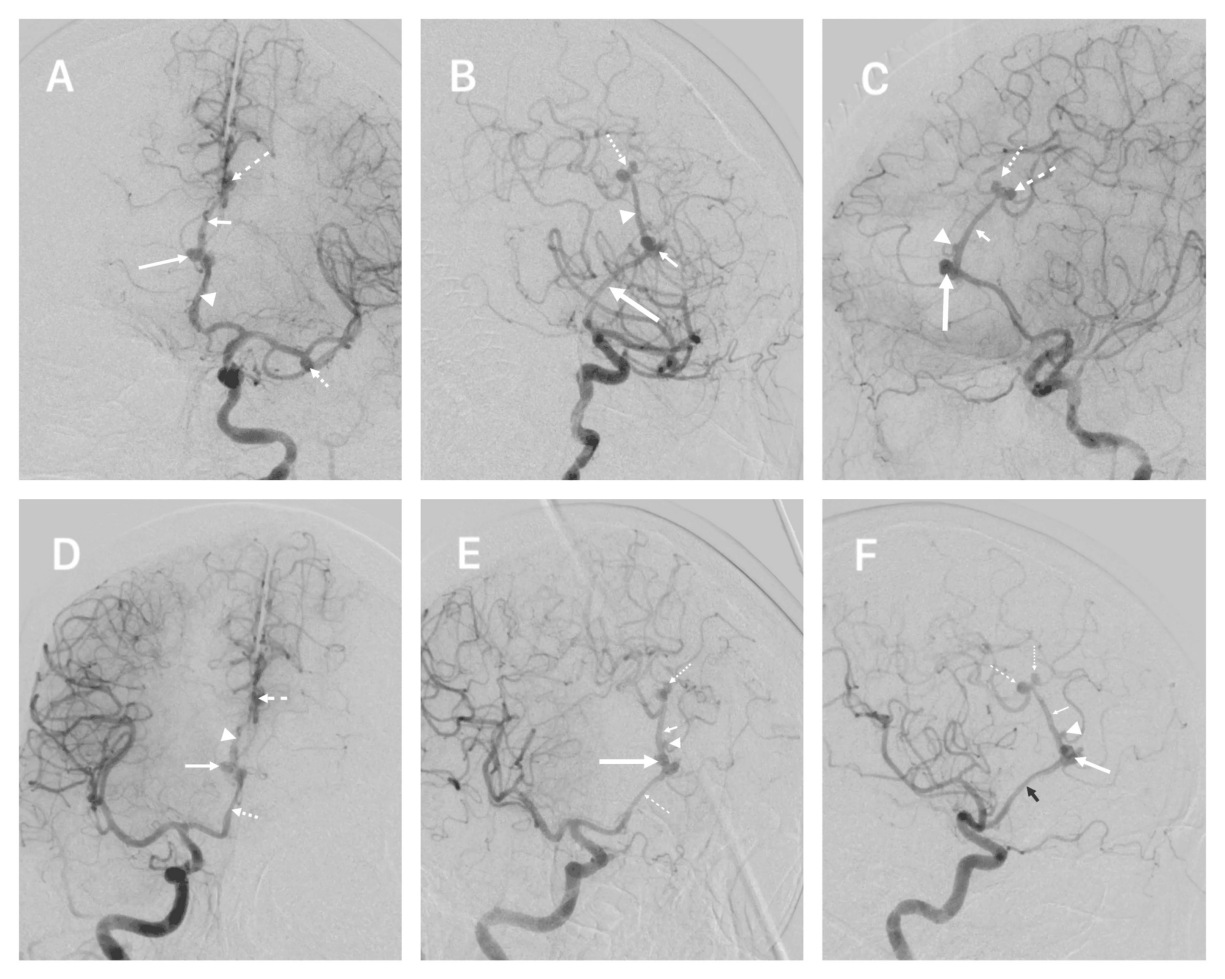
Digital subtracted angiography (A) Left anteroposterior DSA showing an anterior A3 DACAa (long arrow), mirror DACAa (dashed arrow), MCA aneurysm (dotted arrow), azygos variant (arrowhead) and single pericallosal artery variant (short arrow). (B) Left oblique DSA showing an anterior A3 DACAa (short arrow), mirror DACAa (dotted arrow), azygos variant (long arrow), and single pericallosal artery variant (arrowhead). (C) Left lateral DSA showing an anterior A3 DACAa (long arrow), mirror DACAa (dotted and dashed arrows), small A3 DACAa (arrowhead), and single pericallosal artery variant (short arrow). (D) Right anteroposterior DSA showing anterior A3 DACAa (long arrow), mirror DACAa (dashed arrow), azygos variant (dotted arrow), and single pericallosal artery variant (arrowhead). (E) Right oblique DSA showing an anterior A3 DACAa (long arrow), mirror DACAa (dotted arrow), azygos variant (dashed arrow), single pericallosal artery variant (short arrow), and a small A3 DACAa (arrowhead). (F) Right lateral DSA showing an anterior A3 DACAa (large arrow), mirror DACAa (dotted and dashed arrows), small A3 DACAa (arrowhead), and single pericallosal artery variant (small arrow). DSA: digital subtracted angiography, DACAa: distal anterior cerebral artery aneurysm, MCA: middle cerebral artery

A 61-year-old female patient presented to the emergency room in our institution. The patient was in a critical clinical state, intubated, and heavily sedated. The clinical scenario had begun one day before admission, with a sudden thunderclap headache that led to an abrupt loss of consciousness. During the interrogation of her next of kin, it was mentioned that she had a history of unmanaged hypertension for 14 years and did not smoke. During her general and neurological exploration, she was sedated, had bilateral 2 mm constricting pupils, was overweight, had a Glasgow coma score of 3 points, Hunt & Hess score of 5, WFNS of 5 points, and had brainstem reflexes present. Immediate computed tomography (CT) showed a localized anterior interhemispheric hematoma that extended subcallosally (Figure 1A, 1B). Subsequently, the angioCT demonstrated an irregular-shaped anterior A3 DACAa with a width of 9 mm and a broad neck of 7 mm. A second smaller A3 DACAa originating from the emergence of a frontal medial branch 2 mm posterior to the first DACAa, the height was 1.5 mm and a neck of 0.8 mm. The third and fourth were mirror aneurysms originating from the superior A3 segment; the dorsal aneurysm was 3.2 mm in length and had a 2.8 mm neck, and the ventral aneurysm had a length of 3 mm and a neck of 2.6 mm (Figure 1C-1F). Additionally, there was an M1 bifurcation middle cerebral artery aneurysm on her left side that measured 4 mm in length and had a 3.3 mm neck (Figure 1E). The hematoma was contiguous to the larger anterior A3 DACAa. For a better assessment, a digital subtracted angiography (DSA) was performed and confirmed the aneurysms and the presence of an azygos A2 variant with the addition of a post-callosomarginal bifurcation single pericallosal artery trunk (A3) (Figure 2) (Videos 1, 2).

**Video 1:** Left Digital Subtraction Angiography Left Digital Subtraction Angiography with multiple aneurysms.

**Video 2:** Right Digital Subtraction Angiography Left digital subtraction angiography with multiple aneurysms.

**Figure 3:**
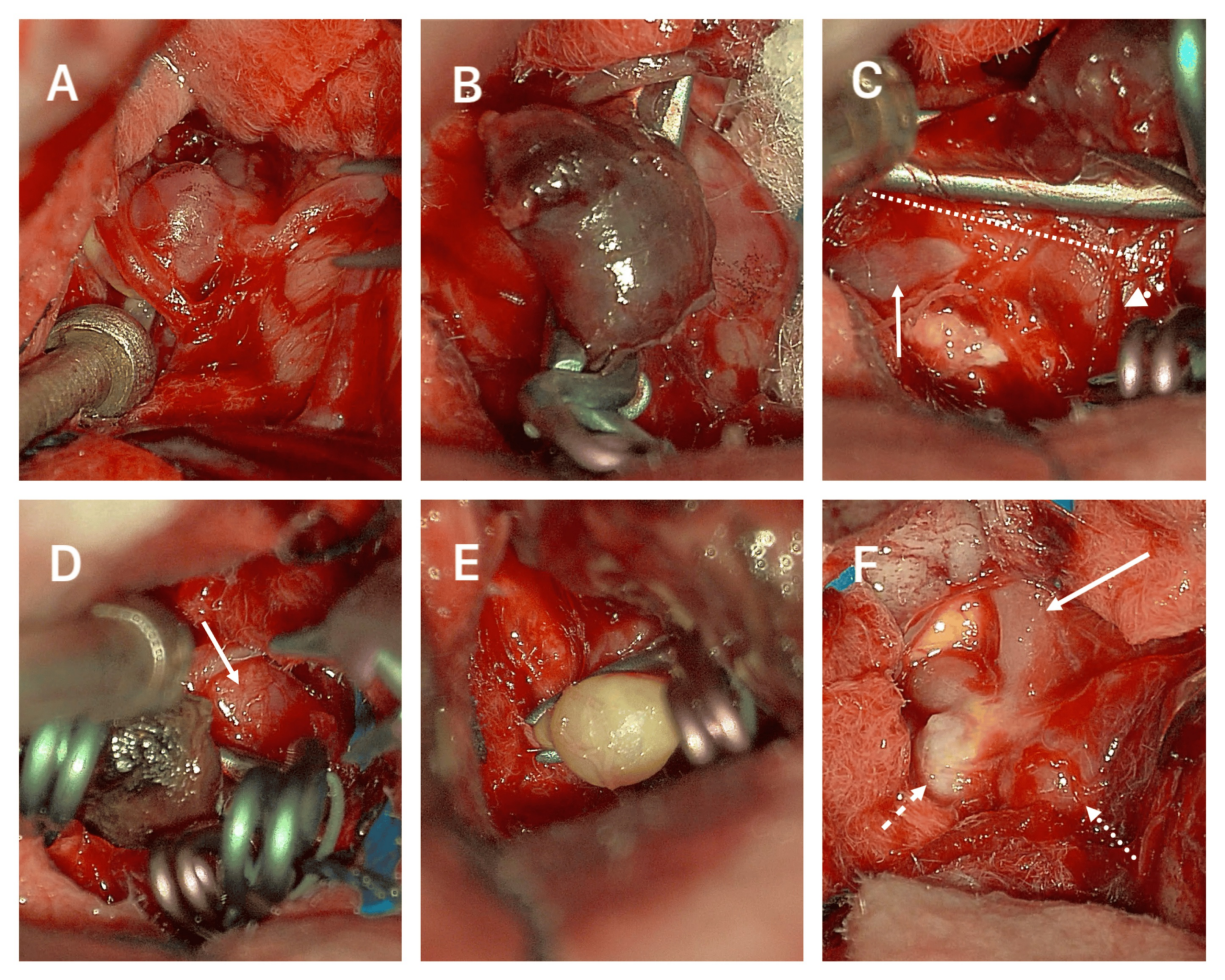
Operative images (A) MCA aneurysm. (B) Large anterior A3 DACAa with a preliminary miniclip. (C) Neck of the large anterior A3 DACAa (dashed line), azygos variant (arrow), single pericallosal artery A3 variant (dotted arrow). (D) Anterior A3 DACAa with multiple clips and a remaining lobule portion (arrow). (E) Small A3 DACAa clipped with significant atheroma plaques. (F) “Kissing” DACAa (dashed and dotted arrows) and the single pericallosal artery variant (arrow). MCA: middle cerebral artery, DACAa: distal anterior cerebral artery aneurysm

The patient was surgically intervened using a left single-staged extended frontotemporal craniotomy that extended from the midline to the root of the zygoma. A subfrontal and transsylvian access was used to clip the MCA aneurysm (Figure 3A). Afterward, we used the anterior interhemispheric access to start a careful dissection anteriorly towards the hematoma and parent vessel A2 segment of the ACA. After removing most of the hematoma, we encountered the anterior A3 DACAa and clipped it using four clips due to the irregular form and broad base (Figure 3B-3D). Posteriorly, between the anterior A3 and the superior A3 segment, an irregular 3 mm aneurysm harboring an atheroma plaque was encountered and clipped (Figure 3E). Finally, following the single A3 pericallosal trunk posteriorly, we encountered the mirror superior DACAa, and each was clipped with a single miniclip (Figure 3F). A Doppler ultrasound examined all distal branches and ACA trunk for permeability.

After surgery, the patient maintained the same critical state. A postoperative angioCT and CT were taken, and adequate clip site and vessel permeability were demonstrated. During the first day, no relevant clinical changes were seen. On the second day, the patient presented a dilated left pupil and required an urgent left decompressive craniectomy (due to cerebral edema, no rebleeding was seen). The pupils eventually returned to 2 mm, and the patient maintained clinical stability for three days. On the sixth day, a control CT was taken due to the lack of clinical improvement seen in the patient. The CT showed a right MCA territory infarct (due to vasospasm) and a left superior temporal gyrus infarct (possibly surgery-related or vasospasm). No late DSA or magnetic resonance angiography (MRA) was performed. The patient presented an unfavorable clinical evolution and eventually died after a month due to hospital-acquired pneumonia.

## Discussion

DACAa are considered any aneurysms that emerge from the A2 to the A5 segments of the ACA [5]. DACAa are an infrequent location for aneurysmal formation and account for a small number of aneurysmal ruptures. They are commonly related to having a broader neck and being small, as regards the parent vessel [1-4]. The origin of the callosomarginal artery within the A3 segment represents the most common site for DACAa in almost 69-82% of the cases; they have been called pericallosal artery-callosomarginal artery junction aneurysms [1,3,4,18,19].

In a study by Lehecka et al., they further divided A3 segments into inferior, anterior, and superior. The inferior A3 aneurysms are situated on the proximal section beneath the corpus callosum, representing 21% of all DACAa cases. The anterior A3 aneurysms are located anterior to the corpus callosum and represent the majority of all DACA mentioned before (callosomarginal bifurcation) and represented 69% of all ruptured and 59% of all unruptured DACAa; our patient presented the ruptured DACAa in this site. Superior A3 aneurysms are found on the distal portion above the genu of the corpus callosum, representing 2% of all ruptured and 0% of all unruptured DACAa [2]. In our case, the patient had two unruptured “kissing” aneurysms in this area. Additionally, our patient had an A3 aneurysm not mentioned in this publication; the aneurysm originated from the bifurcation of a branch between the anterior A3 and superior A3 (Figures 1-3).

DACAa sizes are usually within 5-8 mm [18-21]. More than half of all ruptured and unruptured DACAa represent a size of less than 7 mm. The DACAa between the anterior and the superior DACAa had a larger diameter observed during surgery than the DSA lumen observed preoperatively due to the atheroma plaque in the interior of the aneurysms. In our case, all aneurysms were smaller than 10 mm. The rest of the aneurysms were less than 25 mm. As seen in the literature, all our patients’ DACAa had a broad-based neck and represented a challenge when clipping. Most DACAa have a neck wider than the parent vessel; this was true for most of our patients’ DACAa [1,19].

ACA anomalies developing DACAa is a frequent scenario and is present in a high percentage, ranging from 7-35%, and commonly describe primarily the azygos variation [1,11,18,22,23]. Azygos ACA variation ranges from 0.05% to 1.8% but has a mean prevalence in the normal population of 1.5% in a recent meta-analysis. Azygos is frequently described as initiating posterior to the A1 segment of the ACA, accompanied by a missing ACoA extending through the A2 segment, under the genu, or until the bifurcation of the callosomarginal artery [4,6-8,10,24]. Different terms have been used for the azygos variant: unpaired pericallosal artery, azygos pericallosal artery, unpaired cerebral artery, arteria termatica, and common arterial cerebral trunk [10]. Our patient presented the typical azygos variation and an extremely rare single post-bifurcation pericallosal trunk running along the corpus callosum that bifurcates in the top portion of the genu of the corpus callosum and presents with two saccular superior A3 DACAa. This is a variant that has not been described in previous publications (Figures 1-3). Different terms like unpaired pericallosal artery, azygos pericallosal artery, unpaired cerebral artery, arteria termatica, and common arterial cerebral trunk erroneously depict this variation as an azygos and not the variation seen in our patient [4,6-8,10,12,25-30].

DACAa is knowingly associated with multiple aneurysms (25 to 55%); the most frequent is the MCA [1,9,18,31]. In the Lehecka et al. series, multiple aneurysms were present in 50% of the cases, with 6% including other DACAa. There were a total of 111 aneurysms in 50 patients; 60% of all aneurysms were in MCA, 14% in the ICA, 10% ACoA, 9% in vertebrobasilar artery, and 6% in the DACA [1]. Our patient presented a right MCA aneurysm [1,2].

Once surgical intervention is indicated, having multiple aneurysms may change the approach strategy and necessitate a single-staged craniotomy, a two-staged craniotomy, or a hybrid endovascular intervention. We opted for an extended single-staged frontotemporal craniotomy with interhemispheric and subfrontal-transsylvian access. Also, having subfrontal access before accessing the interhemispheric route could grant us proximal control in both the internal carotid artery (ICA) or bilateral A1 and drainage of cisterns [32,33].

Since severe brain edema and intraparenchymal hematoma were present and there was a need for the resolution of multiple aneurysms and a broad neck of the aneurysms, endovascular treatment was not recommended [1]. For DACAa, open surgery leads to improved aneurysm occlusion and reduced recurrence [34,35]. Endovascular management carries significant risks, including arterial dissection, procedural rupture, inadvertent parent vessel, and incomplete aneurysm treatment occlusion [34,36]. The initial clinical assessment indicated an unfavorable outcome, necessitating urgent intervention; the patient's evolution was anticipated. Unfortunately, the patient developed severe brain edema due to the underlying event, required additional surgical and medical interventions, and ultimately succumbed to a hospital-acquired pneumonia that led to his death. The surgery did not result in any brain contusion.

Our limitations primarily include the lack of institutional fluorescein and indocyanine green; the patency was examined with Doppler ultrasound. Also, our institution is limited to facilitated endovascular therapy, and prolonged waiting times lead to worse outcomes.

## Conclusions

The presence of patients exhibiting a ruptured anterior A3 segment of the DACAa, accompanied by unruptured kissing superior A3 DACAa, as well as an atheromatous DACAa situated between the anterior and superior A3 segments, a total of five multiple aneurysms, a presentation of the uncommon azygos variation, and the presence of a solitary post-bifurcation pericallosal artery trunk, renders each case a rare entity. The combination of all these findings makes this particular case extremely rare. The management of this patient was addressed in a single surgical setting, avoiding multiple interventions. To manage complex cases effectively, it is essential to have a comprehensive strategy that establishes a clear surgical approach while preventing any additional harm to the patient. This particular case showcases a patient with unusual anatomical variations and complex vascular disease that can be treated with a single-stage craniotomy.
